# Simultaneous multiple injection to perform titration and standard addition in mono-segmented flow analysis

**DOI:** 10.1155/S1463924601000104

**Published:** 2001

**Authors:** Margarete Assali, Ivo M. Raimundo, Jr, Ileana Facchin

**Affiliations:** 1 Instituto de Química UNICAMP CP 6154 Campinas São Paulo CEP 13083-970 Brazil; 2 Instituto de Ciências da Saúde UNIP Objetivo Campinas Brazil


Journal of Automated Methods & Management in Chemistry, Vol. 23, No. 3 (May–June 2001) pp. 83–89

### Simultaneous multiple injection to perform titration and standard addition in monosegmented flow analysis

**Margarete Assali[1], Ivo M. Raimundo Jr[1,2] and Ileana Facchin[1,3]**

*[1]Instituto de Química, UNICAMP, CP 6154, CEP 13083-970, Campinas, São Paulo, Brasil; [2]e-mail: ivo@iqm.unicamp.br; [3]permanent address: Instituto de Ciências da Saúde, UNIP, Objetivo, Campinas, Brazil*

*An automated system to perform titration and standard addition in monosegemented flow analysis by employing the simultaneous multiple injection is described. The system was controlled by a PC-AT-386 microcomputer through a home-made parallel interface, employing a diode array spectrophotometer as detector. Software was written in QuickBasic 4.5 to control the system and for data acquisition. A three-way solenoid valve was used in conjunction with a proportional injector to add the titrant solution or the standard solution to the sample, to carry out titration or standard addition, respectively. Only one standard solution was used in each procedure and different quantities of titrant or standard were added to the sample by controlling the time interval in which the solenoid valve was switched on. Titration and standard addition curves similar to those of the manual methods were obtained in both cases, since the sample dispersion was very low due to the air bubbles of the monosegment. The titration system was evaluated through the determination of Fe(II) with a $KMnO_4$ standard solution in pharmaceutical preparations. The standard addition process was assessed by determining Cr(VI) in natural waters and domestic wastewater using the diphenylcarbazide method. The results obtained in both methodologies did not differ significantly from the reference methods at a 95% confidence level.*

### Introduction

Since its introduction in 1985 [1], monosegmented flow analysis (MSFA) has proved useful to perform several different spectrophotometric determinations, in both aqueous [2, 3] and gaseous [4, 5] matrices. As the most flow analysis techniques, MSFA allows high sample throughput, with low consumption of reagents and sample. However, its main characteristic arises from the fact that dispersion of the sample is very low, even for long residence times, since the sample is injected into the system between two air bubbles. As a consequence, sensitive methodologies based on a catalytic reaction [6] and on solvent extraction [7] have been developed by employing MSFA systems.

Concentration gradients are inherent phenomena in FIA [8, 9] and SIA [10] systems, since the sample undergoes dispersion. In these systems, sample dispersion is usually maintained as low as possible to provide sensitive methodologies. However, the gradient concentration profile generated when the sample dispersion is relatively high has frequently been exploited to perform sample dilution [11] and also to implement titration [12–31] and standard addition [32–45] procedures in flow analysis.

Flow injection titration was first described by Ruzicka *et al.* [12] in 1977. A single-line manifold was employed, in which the sample was injected into the titrant carrier fluid and pumped towards a mixing chamber, before reaching the detector. In this system, the peak width is proportional to the concentration of the sample and, therefore, a calibration curve is necessary. This approach has been successfully applied to analyse samples from different matrices [13–15].

Automatic flow titrations based on variation of flow rates of sample and titrant have been shown feasible [16–19]. In such systems, two peristaltic pumps are employed and the sample flow rate is usually maintained constant, while the titrant flow rate is changed, generating a concentration gradient that allows carrying out the determination. Besides the use of two peristaltic pumps, this approach shows a drawback related to pumping tube deterioration. To overcome this problem, an alternated exponential flow has been proposed [20], employing two peristaltic pumps to deliver sample and titrant solutions. The pumping speed of one pump is continuously increased, while the pumping speed of the other one is proportionally decreased. A three-way solenoid valve is employed to alternate sample and titrant pumping between the two pumps, compensating for tube wear.

Triangle-programmed flow titration, proposed by Nagy *et al.* [21], is based on the linear increase of the titrant concentration, followed by its symmetrical decrease. As the sample flow rate is maintained constant, two endpoints are obtained, allowing determination of the sample concentration. This approach has been applied to different systems, some employing a coulometric generation of the titrant [22, 23] and also using a linear volumetric flow gradient [17, 24].

Titration in multisegmented continuous flow systems has been described by Fleet and Ho [25]. In this system, the sample and titrant flow rates are maintained constant while the concentration of the titrant is varied, providing a concentration gradient. The peak width is proportional to the analyte concentration, which is calculated through an analytical calibration curve. Sequential injection analysis has been employed by Sultan *et al.* [26] to implement a spectrophotometric flow titration methodology to determine vitamin C in pharmaceutical products. A Ce(IV) standard solution was used as titrant and a calibration curve was necessary to carry out the analysis.

Although flow titration methodologies, such as those above mentioned, usually employ an analytical curve to determine the concentration of the analyte in the sample, they can be performed without this calibration step. Korn *et al.* [27] have performed acid–base titrations in flow systems employing the concept of binary searching, in which the volumetric fractions of titrant and sample are continually varied until the stoichiometric ratio is reached. This procedure is feasible because the titrant and sample volumes inserted into the analytical path were exactly known. Araújo *et al.* [28] described spectrophotometric and conductometric titration methodologies based on the calibration of the concentration gradient that takes place by injecting the sample in the titrant carrier stream. Since the dispersion of the sample is the same as the solution used in the calibration of the gradient, the concentration of the analyte in the sample can be determined straightforwardly from the titration curve obtained in a single injection.

Recently, different approaches have been proposed to determine the titration end point in automatic monosegmented flow systems, which were constructed by using solenoid valves [29–31]. Martelli *et al.* [29] employed the binary search concept to determine the end-point in a potentiometric titration with NaOH standard solution. In this procedure, the sample volume was constant while the titrant volume was varied according to the successive approximation strategy. The volume of the monosegment was maintained constant, by adding proper aliquots of an inert diluent solution. During the titration, depending on the sample/titrant volume ratio, the potential of the indicator electrode can be higher and/or lower than a preset potential of the end-point. The titrant volume is varied until the preset potential is reached, then the titration is stopped. Honorato *et al.* [30] described the use of the Fibonacci algorithm to conduct spectrophotometric acid–base titrations in a monosegmented flow system to determine the acidity of vinegar. Volumes of the sample and the titrant were always varied after each injection according to the algorithm, maintaining the volume of the monosegment constant. While the titration was carried out, the interval of uncertainty related to the titrant volume was minimized, until a predefined interval was reached, indicating the titration end point. Ganzarolli *et al.* [31] also described an automatic potentiometric titrator to determine strong and weak acids with a standard sodium hydroxide solution. The end-point was determined by injecting several mixtures of sample and titrant in different volumetric ratios, defined according to a successive approximation algorithm.

Methodologies based on standard addition have been widely employed in chemical analysis to overcome drawbacks about matrix effects. Since the procedures are usually time consuming, a great deal of attention has been paid to develop standard addition flow systems, which allow speeding up the analysis [32–47]. Three different approaches have been employed to perform standard addition in flow systems, based on the continuous pumping of sample (reversed FIA) [32–37], continuous pumping of the standard (conventional FIA) [38,39] or on the merging zone process [40–44].

Tyson and collaborators [32–35] proposed a standard addition procedure in FIA with AA detection, in which the sample was continuously pumped towards the nebulizer, generating a steady-state absorbance signal. Discrete aliquots of standard solutions were then injected into the carrier stream, producing positive or negative transient signals whenever the standard concentration was higher or lower than the sample concentration, respectively. Therefore, a calibration curve was obtained and the sample concentration determined by interpolation in the equation of the curve. Israel and Barnes [36], in 1984, used the standard-to-sample injection principle described above to implement a simpler SAM based on only two injections, water (or other solvent matrix) and standard solution. Araújo *et al.* [37] described a flow system for generalized standard addition method (GSAM) based on single standard injection into a sample, pumped continuously towards the detector, to determine sodium, potassium and calcium by flame photometry. Bechmann *et al.* [38] also proposed a flow injection procedure for GSAM with photodiode array detection, where the sample is continuously pumped and merges with a carrier stream into which the standard is injected.

In 1989, Israel and Barnes [39] developed a sample-to-standard addition method also based on two injections, blank and sample, which showed the disadvantage of matching matrix composition into standard carrier composition. Beuchemin [40] employed a variation of the method proposed by Israel and Barnes [39] for ICP-MS. The concentrations of the samples were determined by means of three injections, that is, injection of the sample into a standard blank solution and into a standard (with concentration higher than the sample) carrier solution, and injection of the standard into the blank carrier stream.

The merging zone concept has been also used to implement standard addition in flow injection [41–45]. In this approach, sample and standard are simultaneously injected into two symmetrical lines, which merge at a confluence point, providing the addition of standard to the sample. Zagatto *et al.* [41] described a merging zone flow system for GSAM applied to determination of metals in alloys by ICP-AES. The main disadvantage of this approach is the need to prepare many standard solutions to allow the addition of standards at different concentrations. This drawback was overcome by Giné *et al.* [42], who employed sampling of the dispersed zone to promote standard addition in a merging zone flow manifold using a single standard solution for determination of nitrate in plants. The merging zone approach has been also implemented by employing a three-way solenoid valve-based flow manifold [43] and even further improved by using a single injection of standard and sample [44, 45].

Standard addition methodologies have been also implemented in unsegmented flow [46] and SIA [47] systems. Recently, Campíns-Falcó *et al.* [48] compared the performance of different approaches [35, 36, 38–40] for standard addition in FIA and Lavilla *et al.* [49] studied the applicability of the method proposed by Israel and Barnes [39] with and without the use of a matrix-matched blank for determination of metals in sewage sludge.

As can be seen, flow injection has been widely employed for the implementation of standard addition methodologies. However, these methods can show some limitations that arise from the fact that matrix effects can be altered due to sample dilution and/or differences in sensitivities when measurements are taken in the steady-state condition and during the transient signal. To avoid these problems, a mathematical model is usually derived, which takes into account these factors, allowing for the calculation of the sample concentration.

Brito and Raimundo, Jr [50] introduced the simultaneous multiple injection (SMI) approach in monosegmented flow analysis (MSFA). In the referred approach, a monosegment composed of air/solution 1/solution 2/solution 1/air is injected into the carrier fluid, being homogenized while it is propelled through the system towards the detector. In this case, one of the solutions should be the sample and the volume ratio between solutions 1 and 2 is chosen according to the requirements of the methodology that is being used. For example, in the original work [33], dilution of a blood plasma sample (diluting solution in larger volume) was performed for determination of glucose and addition of reagents (in smaller volume) was used for determination of nitrite in natural waters. Besides these applications, Silva and Pasquini [51] also used the SMI approach to develop a bisegmented flow system for gas analysis.

In the present work, the SMI approach is employed to implement titration and standard addition procedures in MSFA, taking one of the solutions injected into the system as a titrant or as a standard solution, respectively. A titration methodology employing potassium permanganate standard solution was developed to determine iron in pharmaceutical products and the results were compared with the *US Pharmacopoeia* method. Chromium (VI) was determined in natural waters and domestic wastewater by using the diphenylcarbazide method, and compared with the same procedure using a manual method.

### EXPERIMENTAL

### *Reagents and solutions*

Analytical-grade reagents and distilled/de-ionized water were employed to prepare all solutions.

For Fe(II) titration, the $KMnO_4$ standard solution and Zimmermann-Reinhardt reagent were prepared as recommended [52]. The $KMnO_4$ was standardized using a primary standard grade $Na_2C_2O_4$ reagent. A 0.1000 mol $l^{-1}$ Fe(II) solution was prepared from $Fe(NH_4)_2(SO_4)_2$. A 0.1000 mol $l^{-1}$ Ce(IV) standard solution was prepared from $Ce(SO_4)_2$.

For the standard addition procedure, a 10.00 mg $l^{-1}$ Cr(VI) stock standard solution was prepared from $K_2Cr_2O_7$. A 2.0 mol $l^{-1}$ $H_2SO_4$ solution and a 0.25% (w/v) diphenylcarbazide in 25% (v/v) acetic acid solution were also used.

Pharmaceutical preparations and natural water samples were employed without any previous treatment. Wastewater samples were filtered before injections.

### *Monosegmented flow manifold*

The monosegmented flow analyser employed here has been described elsewhere [53]. A home-made diode-array spectrophotometer was employed as detector [54]. An Ismatec MP13GJ-4 peristaltic pump equipped with Tygon® tubes was employed to propel the fluids. A proportional injector [55] was used in conjunction with a three-way solenoid valve (NResearch, 12 V, 80 mA) to perform the sample injection in the titration and standard addition procedures (figure 1). In the titration procedure (figure 1A), loops L3 and L4 (total volume = 265 µl) and loop L5 (50 µl) were employed to inject the titrant and the sample, respectively. In the standard addition procedure (figure 1B), loops L3 and L4 (total volume = 250 µl) and loop L5 (80 µl) were used to inject sample and standard solution, respectively. An 800 mm long and 1.6 mm i.d. PTFE reaction coil was used in both procedures. Software was written in Microsoft QuickBasic 4.5 to control the manifold and data acquisition, allowing the injection of increasing quantities of titrant or standard solutions in the titration and standard addition procedures, respectively.

### *Procedure*

The parameters of the spectrophotometer and the analyser parameters were initially defined by the user. These parameters include the wavelength of detection, the number of injections (up to 15) to obtain the titration or the standard addition curves, the number of replicates for each injection and the time intervals in which the three-way solenoid valve (figure 1) is turned on during the sampling step. In the sampling step, the appropriate loops are initially filled with the 0.1 mol $l^{-1}$ sulphuric acid solution (titration) or de-ionized water (standard addition). Then, the solenoid valve is turned on and the titrant or the standard solution allowed to flow into these loops. Concomitantly, the sampling loop is filled with the respective sample. The time interval in which the solenoid valve is on is increased in each subsequent injection to add to the sample increasing quantities of titrant or standard solution. As soon as the time interval was completed, the sampling valve was automatically switched to the injection position. Once the monosegment had been introduced into the reaction coil, the sampling valve was moved back to the sampling position. A curve of absorbance versus time (time interval in which the valve is on) is plotted in real time on the video screen of the microcomputer.

In the titration, a 0.1 mol $l^{-1}$ sulphuric acid solution was employed as the carrier fluid at a flow rate of 2.4 ml $min^{-1}$. A $7.5\times10^{-4}$ mol $l^{-1}$ $KMnO_4$ solution was injected through loops L3 and L4 (figure 1A). Absorbance measurements were made at 525 nm. Sample solutions were prepared by transferring 1.0 g (±0.1 mg) of the material and 25 ml of the Zimmermann-Reinhardt solution to a 100.0 ml volumetric flask, which was then filled with de-ionized water.

In the standard addition procedure, de-ionized water was used as carrier fluid at 2.0 ml $min^{-1}$. The 10.00 mg $l^{-1}$ Cr(VI) standard solution was injected by employing loop L5 while the water sample was injected

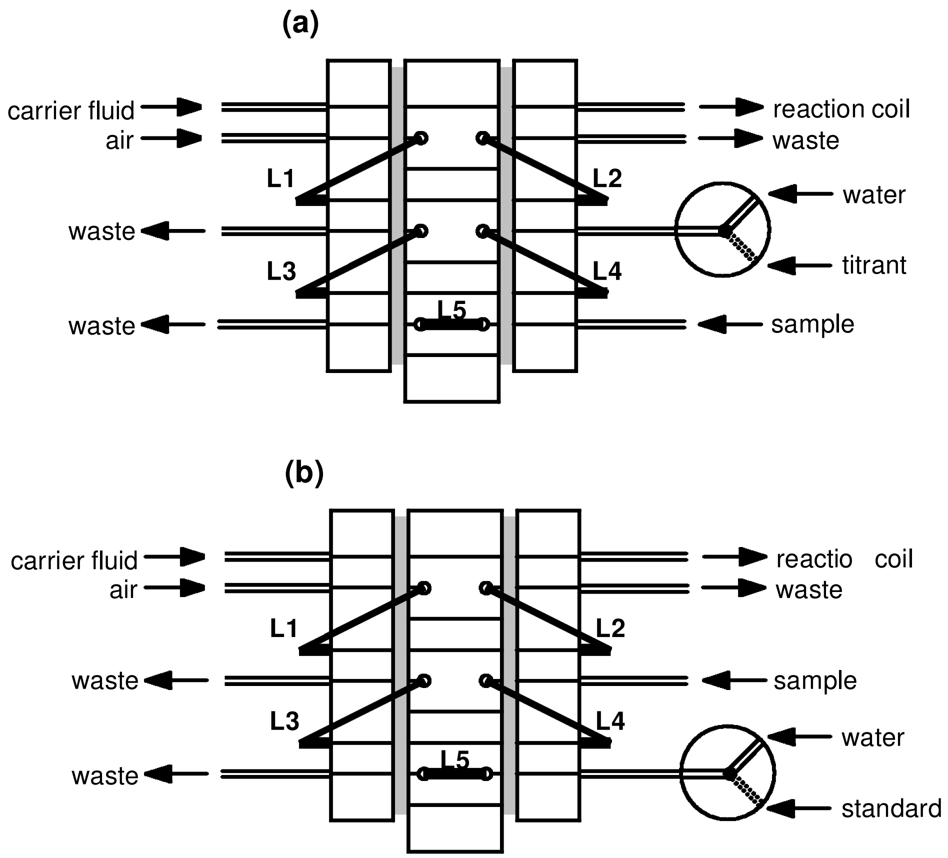

*Figure 1. Sampling valve assembly for (a) titration and (b) standard addition (valve in the sampling position).*

through loops L3 and L4 (figure 1b). After the injection of the monosegment into the reaction coil, the sulphuric acid and diphenylcarbazide reagents were added to the sample at a flow rate of 0.07 and 0.05 ml $min^{-1}$, respectively, by employing an automatic reagent addition module described elsewhere [53]. Absorbance measurements were carried out at 540 nm.

*Calibration of the system*

Since absorbance versus time curves are obtained in both procedures, it is necessary to determine the quantities of titrant or standard solutions added to the sample in each injection. In addition, as the sample is diluted in both procedures, the dilution factor needs to be calculated.

For the titration procedure, loops L3, L4 and L5 are initially filled with a $KMnO_4$ solution, whose concentration ($C_0$) is exactly known. The monosegment is then introduced into the system and the absorbance ($A_0$) is measured. In the subsequent measurements, these loops are initially filled with water. Afterwards, the solenoid valve is switched on and the $KMnO_4$ standard solution is allowed to partially fill loops L3 and L4. After a prefixed time interval ($t_i$), the valve is turned off and the monosegment is injected into the reactor. As the interval increases after each injection, different absorbances ($A_i$) are obtained. The $KMnO_4$ concentration in the monosegment ($C_i$), for each injection, is obtained multiplying the initial concentration of the standard solution ($C_0$) by the absorbance ratio $A_i/A_0$. An analytical curve, $C_i$ versus $t_i$, is then obtained. The dilution of the sample is determined by measuring the absorbance of the monosegmented ($A_s$) when loop L5 (sample) is filled with $KMnO_4$ and loops L3 and L4 are filled with water. The sample dilution factor ($f$) is given by the $A_0/A_s$ ratio.

In the case of the standard addition procedure, the concentration of Cr(VI) in the monosegment and the sample dilution are calculated in a similar manner, using the same $KMnO_4$ standard solution. However, as loops L3 and L4 are for sample and loop L5 is for the Cr(VI) reference solution, that is, the reverse of the titration procedure, the $A_i$ and $A_s$ absorbances are also obtained by filling these loops with $KMnO_4$ and water in an inverse manner. Furthermore, to obtain $A_0$, $A_i$ and $A_s$ values, water is added to the monosegment through the automatic reagent addition module [53] at the same flow rate as reagents are added to the sample during the analysis.

### Results and discussion

The monosegment flow system described here mimics the manual procedures for titration and standard addition, since the sample is confined in between two air bubbles. The system needs to be calibrated initially before performing either of these procedures to determine the quantity of titrant or standard solution added to the sample. Furthermore, the dilution of the sample should be determined to permit the calculation of the analyte concentration in the sample.

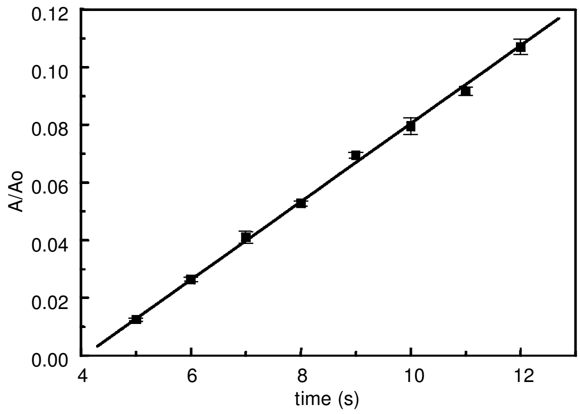

*Figure 2. Analytical calibration curve obtained with the standard addition flow set up (error bars are the SD of three measurements).*

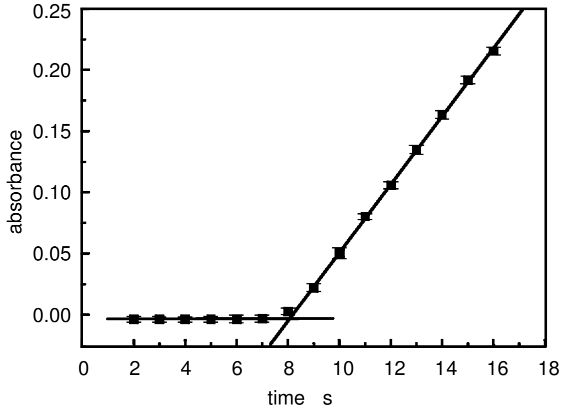

*Figure 3. Titration curve obtained in the titration of a* $5.18 \times 10^{-3}$ *mol l*$^{-1}$ *Fe(II) solution with a* $7.50 \times 10^{-4}$ *mol l*$^{-1}$ *$KMnO_4$ standard solution (error bars are the SD of three measurements).*

The calibration step, as described above, works if Beer's law is strictly obeyed. Therefore, the response of the spectrophotometer was firstly evaluated, showing a linear response up to $7.5 \times 10^{-4}$ mol l$^{-1}$ when a $KMnO_4$ solution was employed. The repeatability and reproducibility of the quantity of solution (titrant or standard) added to the sample depends on the flow rate constancy, pump pulsation and tubing wear, besides the interval in which the solenoid valve is turned on, whose control was done by the microcomputer. Owing to both the dead volume between the solenoid valve and the titrant/standard loop and the flow rate of the titrant/standard solution, at least 2 (titration) or 5 (standard addition) seconds were necessary for the solution to reach the loop. Figure 2 shows an analytical calibration curve obtained with the standard addition flow set-up, indicating the good relationship ($r^2 = 0.9993$) between the time in which the valve is turned on and absorbance, and the good reproducibility of the measurements. The concentration of the standard added to the monosegment can be obtained by multiplying the ratio $A_i/A_0$ by the concentration of the Cr(VI) standard solution. It is worth remembering that a linear response is obtained only if the solution does not completely fill the respective loop. The calibration of the flow titration set-up can be performed by a similar procedure, as described above. Once the system has been calibrated, it can be used straightforwardly, unless some parameter has been changed, such as the volume of the sampling loops, pump tubing or the flow rate.

The SMI approach to implement titration in MSFA was evaluated by employing the determination of Fe(II) with a $KMnO_4$ standard solution. Preliminary experiments showed that a residence >80 s was necessary to complete the reaction, providing a sharp end point. Figure 3 shows a typical titration curve obtained in this condition. As a consequence of the low dispersion, the titration curve profile is similar to those obtained using manual methods. The end-point of the titration is given as the interval in which the solenoid valve was turned on, determined at the intersection of the linear portions before and after the end point. From the analytical calibration curve, similar to those shown in figure 2, the concentration of $KMnO_4$ in the monosegment is readily determined and the concentration of Fe(II) in the sample can be calculated from the reaction stoichiometry and the dilution factor.

*Table 1. Titration of artificial Fe(II) samples by MSFA and by the manual method.*

| $[Fe^{2+}]_{MSFA}$(mol l) | $[Fe^{2+}]_{MSFA}$(mol l) |
|---|---|
| $(1.03 \pm 0.02) \times 10^{-3}$ | $(1.00 \pm 0.02) \times 10^{-3}$ |
| $(5.18 \pm 0.09) \times 10^{-3}$ | $(5.15 \pm 0.03) \times 10^{-3}$ |
| $(1.08 \pm 0.04) \times 10^{-2}$ | $(1.02 \pm 0.002) \times 10^{-2}$ |

Values are the mean ± SD of three replicates.

*Table 2. Determination of Fe(II) in pharmaceutical formulations by MSFA and the* US Pharmacopoeia *[55] methods.*

| *Sample* | *%$Fe^{2+}$ (MSFA)* | *%$Fe^{2+}$ (US Pharmacopoeia)* | *SD* |
|---|---|---|---|
| Iberin[1] | 21.67 ± 0.32 | 19.63 ± 0.26 | 2.03 |
| Sulfato Ferroso Bunker[1] | 12.62 ± 0.97 | 12.28 ± 0.71 | 0.34 |
| Sulfato Ferroso Fontovit[1] | 8.05 ± 0.05 | 7.82 ± 0.03 | 0.23 |
| Fer-in-Sol[2] | 2.28 ± 0.09 | 2.02 ± 0.01 | 0.263 |
| Sulfato Ferroso Bunker[3] | 1.06 ± 0.03 | 0.932 ± 0.02 | 0.13 |

[1]Tablets; [2]oral solution; [3]syrup.

Values are the mean ± SD of three replicates.

The system was first evaluated through the titration of Fe(II) solutions in the concentration range of $1.00 \times 10^{-3}$ to $1.00 \times 10^{-2}$ mol l$^{-1}$ and the results obtained were compared with those of visual titration [52], by employing a $KMnO_4$ standard solution as titrant. These results, shown in table 1, do not differ significantly at a confidence level of 95%, indicating the system is useful to perform flow titration. The system was then applied to the determination of Fe(II) in pharmaceutical formulations, which were also analysed according to the procedure recommended by the *US Pharmacopoeia* [56]. These results, shown in table 2, were statistically compared and no significant differences were observed at a confidence level of 95%.

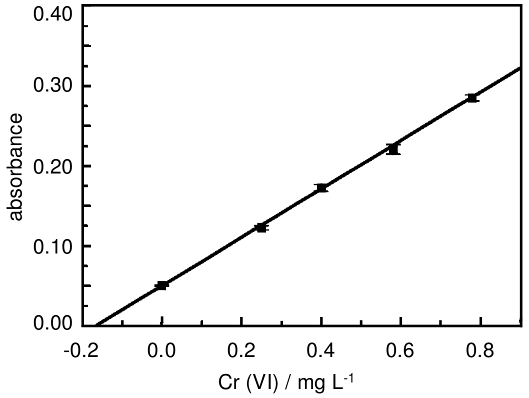

*Figure 4. Curve obtained by additions of Cr(VI) solution to a 0.200 mg l$^{-1}$ Cr(VI) solution (error bars are the SD of three measurements).*

*Table 3. Determination of Cr(VI) in reference solutions.*

| *Cr(VI) added (mg l$^{-1}$)* | *Cr(VI)—MSFA (mg l$^{-1}$)* | *Relative deviation (%)* |
|---|---|---|
| 0.200 | 0.201 ± 0.009 | 0.5 |
| 0.400 | 0.404 ± 0.002 | 0.9 |
| 0.800 | 0.823 ± 0.013 | 2.9 |
| 1.000 | 0.999 ± 0.025 | −0.1 |

Values are the mean ± SD of three replicates.

*Table 4. Recovery tests of Cr(VI) in waterss. Samples were spiked with 0.400 mg l$^{-1}$ metal.*

| | *Recovery (%)* | |
|---|---|---|
| *Water sample* | *MSFA* | *Conventional method* |
| Tap water (Campinas) | 100.3 ± 2.6 | 99.96 ± 2.0 |
| Sea water (Ubatuba) | 103.3 ± 1.3 | 93.5 ± 1.0 |
| Pretinho River (Amazon) | 108.2 ± 1.8 | 78.9 ± 0.7 |
| Pond 1 (UNICAMP) | 104.0 ± 4.7 | 102.04 ± 1.0 |
| Pond 2 (UNICAMP) | 102.4 ± 1.0 | 101.49 ± 1.0 |

Values are the mean ± SD of three replicates.

The experimental conditions employed here allowed a sampling frequency of 120 samples per hour, which means 15 determination per hour, considering that the end-point can be determined from an eight-point titration curve, with a sample consumption of 1 ml.

The determination of Cr(VI) in different water samples was employed to assess the system for the standard addition methodology. In this case, loop L5 was used to add the reagent to minimize sample dilution and also evaluate the addition of small volumes of solution. Figure 4 shows the curve obtained by the addition of standards to a 0.200 mg l$^{-1}$ Cr(VI) reference solution. As can be seen, the repeatability is good and the Cr(VI) concentration can be obtained from the extrapolation of the curve, as in the manual method. However, the value obtained must be corrected considering the dilution undergone by the sample, as a result of the addition of the volume of L5 (standard loop) to the volume of L3 + L4 (sample loop) loops. Table 3 shows the values obtained in the determination of Cr(VI) in artificial samples, containing different concentrations of the metal. The results obtained are in agreement with the expected value, suggesting the feasibility of the methodology.

As the concentrations of Cr(VI) in water samples were lower than the detection limit of the DPC method, all samples were spiked with 0.40 mg l$^{-1}$ of the metal to permit the evaluation of the system. Results obtained are listed in table 4 and compared with those obtained with the calibration curve method. As can be noted, the standard addition procedure can provide better results, mainly for more complex matrices, such as sea water and river water.

A sampling frequency of 72 samples per hour was achieved with the experimental conditions employed, which means the determination of Cr(VI) in ~15 samples per hour, considering that five measurements are necessary to obtain a standard addition curve. The sample consumption was 0.5 ml per determination, which is very low when compared with methodologies based on the reversed-FIA approach, where sample is continuously pumped [32–35, 37, 38]. Furthermore, as the measurements are made in the absence of concentration gradients, because sample is maintained between two air bubbles, the drawbacks regarding the sensitivity differences due to sample/standard dispersion are readily overcome.

### Conclusions

The simultaneous multiple injection approach has been demonstrated as a feasible alternative to implement titration and standard addition procedures, employing MSFA. The proposed system presents as its main advantage a similarity with the manual methods, that is, the monosegment can be considered a microvolumetric flask. As a consequence, data are easily treated and complex equations are not necessary, since sample dispersion is minimal. Moreover, the SMA-MSFA system also provides the advantages of flow methodologies, such as low consumption of sample and reagents and high sample throughput.

### Acknowledgements

The authors kindly acknowledge Professor C. Pasquini and Dr J. J. R. Rohwedder for helpful discussions, Professor C. H. Collins for revision of the manuscript, and the Conselho Nacional de Desenvolvimento Científico e Tecnológico (CNPq, Brazil) for financial support and for the fellowship to M.A.

### References

1. Pasquini, C. and Oliveira, W. A., *Analytical Chemistry*, **57**, 1985, 2575.

2. Smiderle, M., Reis, B. F. and Rocha, F. R. P., *Analytica Chimica Acta*, **386**, 1999, 129.
3. Nogueira, A. R. A., Brienza, S. M. B., Zagatto, E. A. G., Lima, J. L. F. C. and Araújo, A. N., *Analytica Chimica Acta*, **276**, 1993, 121.
4. Silva, M. C. H. and Pasquini, C., *Analytica Chimica Acta*, **349**, 1997, 377.
5. Silva, M. C. H. and Pasquini, C., *Analytica Chimica Acta*, **393**, 1993, 121.
6. Andrade, J. C., Eiras, S. P. and Bruns, R. E., *Analyst*, **118**, 1993, 213.
7. Facchin, I. and Pasquini, C., *Analytica Chimica Acta*, **308**, 1995, 231.
8. Ruzicka, J. and Hansen, E. H., *Analytica Chimica Acta*, **78**, 1975, 145.
9. Stewart, K. K., Beecher, G. R. and Hare, P. E., *Analytical Biochemistry*, **70**, 1976, 167.
10. Ruzicka, J. and Marshall, G. D., *Analytica Chimica Acta*, **237**, 1990, 329.
11. Jorgensen, U. V., Nielsen, S. and Hansen, E. H., *Analytical Letters*, **31**, 1998, 2181.
12. Ruzicka, J., Hansen, E. H. and Mosback, H., *Analytica Chimica Acta*, **91**, 1977, 87.
13. Fuhrmann, B. and Spohn, U., *Journal of Automatic Chemistry*, **15**, 1993, 209.
14. Couto, C. M. C. M., Lima, J. L. F. C. and Montenegro, M. C. B. S. M., *Analusis*, **26**, 1998, 182.
15. Conceição, A. C., Santos, M. M. C., Gonçalves, M. L. S. S. and Santos, F. J. V., *Talanta*, **50**, 2000, 1245.
16. Yarnitzky, C. N., Klein, N. and Cohen, O., *Talanta*, **40**, 1993, 1937.
17. García, I. L., Viñas, P., Campillo, N. and Córdoba, M. H., *Analytica Chimica Acta*, **308**, 1995, 67.
18. Marcos, J., Ríos, A. and Valcárcel, M., *Analytica Chimica Acta*, **261**, 1992, 489.
19. Marcos, J., Ríos, A. and Valcárcel, M., *Analytica Chimica Acta*, **261**, 1992, 495.
20. Yarnitzky, C. N., Klein, N. and Cohen, O., *Talanta*, **40**, 1993, 1937.
21. Nagy, G., Tóth, K. and Pungor, E., *Analytical Chemistry*, **47**, 1975, 1460.
22. Nagy, G., Tóth, K. and Pungor, E., *Analytical Chemistry*, **26**, 1979, 1143.
23. Nagy, G., Tóth, K. and Pungor, E., *Analytical Chemistry*, **46**, 1979, 5.
24. Yarnitzky, C. N., Klein, N. and Cohen, O., *Instrumentation Science Technology*, **23**, 1995, 91.
25. Fleet, B. and Ho, A. Y. W., *Analytical Chemistry*, **46**, 1974, 9.
26. Sultan, S. M., Hassan, Y. A. M. and Ibrahim, K. E. E., *Analyst*, **124**, 1999, 917.
27. Korn, M., Gouveia, L. F. B. P., Oliveira, E. and Reis, B. F., *Analytica Chimica Acta*, **313**, 1995, 177.
28. Araújo, M. C. U., Santos, A. V., Honorato, R. S. and Pasquini, C., *Journal of Automatic Chemistry*, **19**, 1997, 157.
29. Martelli, P. B., Reis, B. F., Korn, M. and Lima, J. L. F. C., *Analytica Chimica Acta*, **387**, 1999, 165.
30. Honorato, R. S., Araújo, M. C. U., Veras, G., Zagatto, E. A. G., Lapa, R. A. S. and Lima, J. L. F. C., *Analytical Science*, **15**, 1999, 14.
31. Ganzarolli, E. M., Lehmkuhl, A., Queiroz, R. R. R. and Souza, I.G., *Química Nova*, **22**, 1999, 53.
32. Tyson, J. F. and Idris, A. B., *The Analyst*, **106**, 1981, 1125.
33. Tyson, J. F., Appleton, J. M. H. and Idris, A. B., *Analyst*, **108**, 1983, 153.
34. Tyson, J. F., Appleton, J. M. H. and Idris, A. B., *Analytica Chimica Acta*, **145**, 1983, 159.
35. Tyson, J. F. and Idris, A. B., *Analyst*, **109**, 1984, 23.
36. Israel, Y. and Barnes, R. M., *Analytical Chemistry*, **56**, 1984, 1186.
37. Araújo, M. C. U., Pasquini, C., Bruns, R. E. and Zagatto, E. A. G., *Analytica Chimica Acta*, **171**, 1985, 337.
38. Bechmann, I. E., Norgaard, L. and Ridder, C., *Analytica Chimica Acta*, **304**, 1995, 229.
39. Israel, Y. and Barnes, R. M., *The Analyst*, **114**, 1989, 843.
40. Beauchemin, D., *Analytical Chemistry*, **67**, 1995, 1553.
41. Zagatto, E. A. G., Jacintho, A. O., Krug, F. J., Reis, B. F., Bruns, R. E. and Araújo, M. C. U., *Analytica Chimica Acta*, **145**, 1983, 169.
42. Giné, M. F., Reis, B. F., Zagatto, E. A. G., Krug, F. J. and Jacintho, A. O., *Analytica Chimica Acta*, **155**, 1983, 131.
43. Reis, B. F., Giné, M. F., Krug, F. J. and Bergamin, Fo., H., *Journal of Analytical Atomic Spectrometry*, **7**, 1992, 865.
44. Silva, E. C., Araújo, M. C. U., Honorato, R. S., Lima, J. L. F. C., Zagatto, E. A. G. and Brienza, S. M. B. , *Analytica Chimica Acta*, **319**, 1996, 153.
45. Silva, E. C., Martins, V. L., Araújo, A.F. and Araújo, M. C. U., *Analytical Science*, **15**, 1999, 1235.
46. Agudo, M., Rios, A. and Valcárcel, M., *Analytica Chimica Acta*, **308**, 1995, 77.
47. Baron, A., Guzmán, M., Ruzicka, J. and Christian, G. D., *Analyst*, **117**, 1992, 1839.
48. Campíns-Falcó, P., Bosch-Reig, F. and Blasco-Gómez, F., *Analytica Chimica Acta*, **379**, 1999, 89.
49. Lavilla, I., Perez-Cid, B. and Bendicho, C., *Analytica Chimica Acta*, **381**, 1999, 297.
50. Brito, V. O. and Raimundo Jr, I. M., *Analytica Chimica Acta*, **371**, 1998, 317.
51. Silva, M. C. H. and Pasquini, C., *Analytica Chimica Acta*, **393**, 1999, 121.
52. Skoog, D. A., West, D. M. and Holler, F. J., *Fundamentals of Analytical Chemistry*, 6th edn (Orlando: Saunders College Publishing, 1992).
53. Raimundo Jr., I. M. and Pasquini, C, *The Analyst*, **122**, 1997, 1039.
54. Raimundo Jr., I. M. and Pasquini, C., *Journal of Automatic Chemistry*, **15**, 1993, 227.
55. Bergamin Fo., H., Medeiros, J. X., Reis, B. F. and Zagatto, E. A. G., *Analytica Chimica Acta*, **101**, 1978, 9.
56. *The US Pharmacopoeia: The National Formulary*, 3rd edn (Rockville: US Pharmacopoeia Convention, 1985).

